# Workflow interruptions and mental workload in hospital pediatricians: an observational study

**DOI:** 10.1186/1472-6963-14-433

**Published:** 2014-09-24

**Authors:** Matthias Weigl, Andreas Müller, Peter Angerer, Florian Hoffmann

**Affiliations:** Institute and Outpatient Clinic for Occupational, Social, and Environmental Medicine, Ludwig-Maximilians-University, Munich, Germany; Institute for Occupational and Social Medicine, University of Düsseldorf, Düsseldorf, Germany; Dr. von Hauner University Children’s Hospital, Ludwig-Maximilians-University, Munich, Germany

**Keywords:** Workflow interruptions, Workload, Hospital pediatricians, Observation, Work life

## Abstract

**Background:**

Pediatricians’ workload is increasingly thought to affect pediatricians’ quality of work life and patient safety. Workflow interruptions are a frequent stressor in clinical work, impeding clinicians’ attention and contributing to clinical malpractice. We aimed to investigate prospective associations of workflow interruptions with multiple dimensions of mental workload in pediatricians during clinical day shifts.

**Methods:**

In an Academic Children’s Hospital a prospective study of 28 full shift observations was conducted among pediatricians providing ward coverage. The prevalence of workflow interruptions was based on expert observation using a validated observation instrument. Concurrently, Pediatricians’ workload ratings were assessed with three workload dimensions of the well-validated NASA-Task Load Index: mental demands, effort, and frustration.

**Results:**

Observed pediatricians were, on average, disrupted 4.7 times per hour. Most frequent were interruptions by colleagues (30.2%), nursing staff (29.7%), and by telephone/beeper calls (16.3%). Interruption measures were correlated with two workload outcomes of interest: frequent workflow interruptions were related to less cognitive demands, but frequent interruptions were associated with increased frustration. With regard to single sources, interruptions by colleagues showed the strongest associations to workload.

**Conclusions:**

The findings provide insights into specific pathways between different types of interruptions and pediatricians’ mental workload. These findings suggest further research and yield a number of work and organization re-design suggestions for pediatric care.

## Background

The hospital work environment creates significant potential for workflow interruptions and distractions from a large range of sources [1–5]. We consider clinicians’ workflow interruptions as an intrusion event of an unplanned and unscheduled task, causing a discontinuation of tasks, distraction that causes a noticeable break, or task switch behavior [6]. On the contrary, multitasking activities are carried out in a timely concurrent manner and pediatricians frequently switch between simultaneous task demands [1, 6, 7]. Frequent workflow interruptions are associated with increased psychophysical stress as well as with detrimental quality and clinical safety [8–10]. Interruptive hospital environments are conducive to several limitations in staff’s clinical performance, such as increased surgical errors [11], unfocused clinical procedures, multitasking [6], increased registration errors [12], and medication errors [10]. Workflow interruptions impede health professionals’ attention, task execution and completion, and decision making [1, 9, 13, 14].

Mental workload is defined as an individual’s “costs to achieve a particular level of performance (…) it emerges from the interaction between the requirements of a task, the circumstances under which it is performed, and the skills, and perceptions” [15]. Mental workload intervenes between clinical demands and healthcare staff performance [16, 17]; and increased subjective workload is associated with detrimental clinical performance [16, 18].

Concerning the impact of workflow disruptions on clinicians’ mental demands, a recent study showed that frequent workflow interruptions are associated with increased mental workload in ward physicians [19]. Interruptive clinical environments can greatly add to the psychophysical demands that are inherent to medical care particularly through increasing mental demands on pediatricians [20]. This may affect workload, fatigue, stress, and frustration [21] with possible negative effects for pediatricians’ performance and patient safety outcomes [22].

Despite growing interest in the effects of workflow interruptions in pediatric care, several gaps remain in the evidence base. First, studies investigating the prevalence of interruptions in pediatric care are scarce. To the best of our knowledge, only one study assessed interruptions in pediatric residents’ work routines [23]. Secondly, we found no studies in pediatric care directly linking interruption events faced by pediatricians to individual endpoints in terms of mental workload or stress. Specifically, field studies addressing the mental implications of interruptions events are missing [24, 25]. Thirdly, research is needed to capture the complex effects of workflow interruptions on mental workload [26]. Workload consists of different, somewhat independent dimensions that represent the “mental workload” experienced by pediatricians performing clinical work [27]. Various dimensions of mental workload may be differently related to different sources of interruptions. However, investigating component ratings of physicians’ workload can help investigators pinpoint the source of workload and derive tailored improvement strategies [27]. In this study we sought to address these limitations by drawing on a sample of ward pediatricians’ and aimed to:

1. assess the prevalence of workflow interruptions and the extent of different dimensions of mental workload during pediatricians’ full day shifts;
2. investigate associations between observed workflow interruptions and different dimensions of pediatricians’ mental workload.

## Method

### Study setting and sample

The study was conducted in a 150-bed pediatric clinic of an academic children’s hospital in South Germany and was part of an intervention on pediatricians’ work conditions [28]. Two similar inpatient wards that provide care for all areas of internal pediatric diseases with main focus on gastrointestinal, renal, infectious, and metabolic complications were included. The observations included pediatricians assigned to their ward throughout the entire shift. Overall, nine ward pediatricians of the two internal pediatric wards met inclusion criteria (i.e., pediatricians undergoing specialty training, working permanently during observation period on respective wards, working in patient contact). To limit bias due to inexperience we excluded pediatricians who were working less than four months on the observed wards.

Overall, 28 randomly selected, full shift observations were conducted, in which 7 pediatricians participated: N = 6 female, 85.7%. Each participating pediatrician was observed for at least three full-day shifts to diminish bias due to occasional daily events. Four pediatricians were working on junior-entry-level positions and three pediatricians were working on senior specialist level, which included supervisory duties. Two physicians were not on duty during the randomly selected observations.

During the observations no patient-related information was collected. Pediatricians’ participation was voluntary and they were informed prior to the study commencing, and consent was obtained at least one day before the scheduled observation. This study is part of a larger research program on pediatricians’ work conditions and care quality.

The study was conducted with the approval of the Ethics Committee of the Medical Faculty of Munich University (No. 124/07).

### Design and observation procedure

A prospective, observational design was applied, combining extended structured expert observations and self-report measures [7, 19]. Expert observations in healthcare with standardized measures are an appropriate approach to identify workflow interruptions in clinicians’ work [9, 25, 29]. Full-shift observations of clinicians’ daily work routines enhance internal and external validity of observational results [3, 29]. A trained observer with a medical background (Doctoral Student in Occupational Medicine) shadowed the pediatricians throughout the entire day shift. Prior to the study, the observer was trained on site. Pilot observations were conducted on wards to become familiar with the environment. Additionally double observations with an experienced observer (first author: M. Weigl) were carried out to discuss potential problems in classifying observed interruption events. Finally, observer agreement was assessed during four participant observations. The Kappa-coefficient was 0.63 what indicates substantial inter-rater agreement and tool’s reliability [28].

Mental workload ratings were collected with short questionnaires. Pediatricians reported twice on different dimensions of their mental workload: (i) ‘morning’ , the questionnaires were administered by the observer at half-time of the shift (usually before starting lunch break; or half way through the shift if the doctor omitted lunch break); (ii) ‘afternoon’ , at shift’s end when pediatricians were finished with work. Each time they were requested to estimate their workload in relation to their work over the previous observed part of the shift. This approach allows linking observed interruption events and clinician’s workload reports [19, 30].

### Measures: pediatricians’ workflow interruptions and mental workload

In order to identify pediatricians’ workflow interruptions an established and reliable observational tool was applied [19, 29]. The detailed coding scheme and coding rules to operationalize work interruptions are described elsewhere [29]. Ten sources of pediatricians’ workflow interruption were identified [19, 29]: (1) interruptions by fellow pediatricians; (2) by nursing staff; (3) by telephone/beeper; (4) by patients; (5) by patients’ relatives; (6) by any other person or employee; (7) interruptions due to equipment or technical malfunctions (i.e., equipment dysfunctions or technical malfunctions); (8) Information impediments (i.e., necessary work information unavailable); (9) Waiting time, and (10) Motor or physical impediments (e.g., noise, confined space for moving, additional physical strength in moving heavy patients). Impediments (8–10) are considered as a special subset of workflow interruptions that force doctors to stop the current activity to turn their attention to a disruptive incident and thus aggravate or delay task performance [31]. Pilot reliability tests using parallel observations supported tools reliability prior to the main study.

Pediatricians’ mental workload: An abbreviated three item measure taken from the most validated subjective task load measure was applied, the NASA Task Load Index (TLX) [15]. The NASA-TLX serves as a multidimensional rating technique for the subjective assessment of workload [15, 32]. It is widely used and has been shown applicable to clinicians’ work in various hospital environments [17, 30, 32]. Individual subscale analyses based on pediatricians’ component workload ratings is a feasible option to address particular sources of pediatricians’ workload [27]. Three items from the NASA-TLX were selected for this study: Mental demands (with the instruction “How mentally demanding was your work during the observed period?”), Effort (“How hard did you have to work to accomplish your level of performance?”), and Frustration (“How insecure, discouraged, irritated, stressed, and annoyed were you?”). The scale ranged from 0 (“very low”) to 100 (“very high”).

Further work-related and procedural information was collected: Start and end of morning and afternoon observation period, as workload was assessed at the end of both time periods.

### Analysis

All study data were checked for errors and implausible values (i.e., caused through typing errors). This was established with double entry of raw observational data and check for complete match of the data. The different sources of workflow interruption were checked for overall frequency. The prevalence of observed interruptions was weighted for observation time (to compute interruption rates). Workload reports were tested for mean differences among the three dimensions with t-test. Pearson Correlation analyses to check for relationships among the three workload dimensions and mean differences test (ANOVA) between morning and afternoon observation periods were conducted. In order to investigate associations between the different sources of workflow interruption and pediatricians’ workload ratings we applied linear regression analyses. For each association between an interruption category and reported workload dimension we conducted individual regression analyses. To adjust for potential multi-testing, p-Values for significance were corrected for the number of comparisons using Sidak correction factor (α = .05, number of comparisons = 12, mean correlation among outcome variables = .30, and degrees of freedom = 55). Thus, the threshold for significant findings was adjusted downward to α’ = .012. All analyses were performed using SPSS 20.0.

## Results

Overall, n = 28 participant expert observations were conducted with a duration of 286 hours, and 29 minutes (17,189.42 minutes). The average shift duration was about 10 hours, 13 minutes, and 55 seconds; SD = 0:34:09 (hh:mm:ss), Range 8:55:45 – 11:14:59). Concurrently, all observed pediatricians completed a self-rating twice for each observation - morning and afternoon - resulting in 56 workload evaluations.

### Frequency of pediatricians’ workflow interruptions

During the full-shift observations 1353 workflow interruptions were recorded. Table 1 reports how often each of the different interruptions was observed within an observed working hour on average:

**Table 1:** **Frequency of observed workflow interruptions in ward pediatricians**

		Observed workflow interruptions (n, %)		Interruptions per hour (Mean)	
Overall		1353 (100%)		4.72	
Categories	Workflow interruption by …				
Colleague interruptions	… fellow Pediatricians	1031 (76.2)	408 (30.16)	3.60	1.42
	… nursing staff		402 (29.71)		1.40
	… telephone/beeper		221 (16.33)		0.77
Interruption by others	… patients	163 (12.0)	9 (0.67)	0.57	0.03
	… patients’ relatives		35 (2.59)		0.12
	… by any other person		114 (8.43)		0.40
	… due to equipment/ technical malfunctions		5 (0.37)		0.02
Impediments/Delays	Information impediments	159 (11.75)	13 (0.96)	0.55	0.05
	Waiting time		86 (6.36)		0.30
	Motor impediments		60 (4.43)		0.21

Note: 56 half shift observations; Overall Observation Time: 286 h, 29 min, 42 sec; n number of interruptions.

Of all observed categorized work interruptions, most were caused by colleagues in the immediate working environment (n = 1031, 76.2%). The minor share was almost equally attributed to interruptions by others (n = 163, 12.0%) and impediments/delays (n = 159, 11.75%). Regarding the single sources of workflow interruption, interruptions by fellow pediatricians (n = 408, 30.16%) and by ward’s nursing staff occurred most frequently (n = 402, 29.71%), followed by telephone/beeper interruptions (n = 221, 16.33%). After adjusting for observation time, on average 4.72 workflow interruptions per hour were observed. We did not find a significant difference between observed workflow interruptions during morning (M = 4.57, SD = 1.82) and afternoon periods (M = 4.85, SD = 2.48): F(df = 1) = .23, p = .63.

### Pediatricians’ mental workload during observed shifts

Table 2 presents the average reports of pediatricians’ mental workload dimensions. Overall all three dimensions were within a medium scale range. However, the standard deviation values showed strong variance across the single reports:

**Table 2:** **Dimensions of pediatricians’ reported workload**

		Overall		Time of observation		
***Workload dimensions***				Morning	Afternoon	Significance
	N	M (SD)	Range (Min; Max)	M (SD)	M (SD)	F; p
Mental demands	56	44.3 (17.7)	13; 74	39.2 (17.49)	49.4 (16.7)	4.9; **.03**
Effort	56	49.3 (17.4)	16; 86	44.5 (17.72)	54.1 (16.0)	4.5; **.04**
Frustration	56	46.1 (24.4)	3; 96	42.7 (22.59)	49.5 (26.1)	1.1; .30

Note: Scale Range 0 very low, 100 very high. N Number of half-shift observations. bolded if p <.05.

We compared the mean of all three mental load dimensions: the reported mental demands were almost lower than the effort during the observed periods: ΔM = -4.98, t(df = 55) = 1.94, p = .06. Frustration did not significantly differ between the two other dimensions.

Additionally, we determined the difference between the reported workload dimensions during the morning and afternoon observations. Table 2 shows that Mental Demands and Effort were remarkably higher during the afternoon period than during the morning; reported Frustration was stable across the day shift.

To check for the interrelatedness of the three workload dimensions we then conducted correlation analyses. Mental demands showed a significant correlation to Efforts (r = 0.40, p = .002) but not to Frustration (r = -0.19, p = .14). Efforts were positively correlated with Frustration, r = .27, p = .049).

### Associations of workflow interruptions and pediatricians’ mental workload

To examine the impact of observed workflow interruptions on pediatricians’ workload ratings we conducted regression analyses. We controlled for time of observation because it showed in two out of three workload dimensions meaningful associations with pediatricians’ workload ratings (cf. to Table 2). Table 3 presents the regression estimates of the single analyses:

**Table 3:** **Association of overall and categorized number of workflow interruptions on pediatricians’ workload (Regression Results)**

	***Dependent variables: dimensions of mental workload***		
	Mental demands	Effort	Frustration
***Predictors***	β; p	β; p	β; p
Overall workflow interruptions	**-.40; .002**	-.08; .567	**.37; .005**
Colleague interruptions	**-.37; .004**	-.12; .382	.33; .014
Interruptions by others	-.20; .135	.14; .281	.21; .129
Impediments / Delays	-.24; .066	-.06; .631	.24; .082

Note: N = 56; β standardized regression coefficient; controlled for time of the day, control variable not displayed; bolded if p below adjusted significance level: p < 0.012 (Sidak correction procedure).

The estimation of the prospective impact of the overall frequency of the workflow interruptions showed significant results (cf., Table 3): frequent interruptions were associated with less mental demands above the contribution of the control variable (β = -0.40; p < 0.01). In contrast, frustration was significantly higher with more frequent workflow interruptions: β = 0.37; p < 0.01).

With regard to the impact of the different sources of workflow interruption we observed a negative relationship between colleague interruptions and mental demands: β = -0.37; p = 0.004. Observed impediments and delays showed a substantial but non-significant association to pediatricians’ reported mental demands. Similarly, colleague interruptions and impediments/delays were associated with increased frustration but did not reach significance.

## Discussion

Our study set out to investigate associations between workflow interruptions and different dimensions of workload in pediatricians. Overall, pediatricians were interrupted on average 4.7 times per hour. This underlines previous findings that clinicians act within a work environment with a high potential for distracting events [3, 29, 33]. In comparison to other observational studies, pediatricians working on ward coverage are interrupted with a similar frequency as their colleagues in other specialties [3, 29]. A closer look revealed that interruptions by colleagues were by far most frequent, particularly caused by fellow pediatricians, nursing staff, and telephone or beeper calls [29, 30, 34]. This verifies the inherent demand for intra- and inter-professional communication in hospital physicians’ work [3, 24, 25]. Pediatricians’ average workload reports were moderate but varied to a greater extent throughout the observations what is attributable to the unpredictable nature of clinical workflow [32].

Furthermore, this study aimed to examine potential influences of workflow interruptions on multiple types of pediatricians’ workload (aim 2). For the overall number of workflow interruptions we observed differential associations to mental work dimensions: frequent workflow disruptions were associated with reduced mental demands whereas interruptions increased pediatricians’ reported frustration and stress. Concerning the mental demands reports we assume that frequent disruptions might deter physicians from demanding clinical patients and tasks, which require more complex thought and decision-making. But this assumption deserves further empirical clarification. Our argument is also in line with the missing effect of workflow interruptions on pediatricians’ effort. In the observed sample, frequent workflow disruptions did not trigger additional effort to accomplish the level of performance. We assume that pediatricians’ frustration and stress is intensified by frequent additional disruptive events and requirements. Consequently, clinicians who need to resume interrupted tasks repeatedly feel discouraged, annoyed, and dissatisfied. We also assume that frequent workflow interruptions are due to disruptive and inefficient work practices and internal ward organization. Fragmented workflow may decrease pediatricians’ tolerance for dealing with challenging clinical issues that deserve undivided attention.

Concerning the effects on mental demands there is post hoc also another explanation possible: the content of the interruption might have a positive effect on the current task at hand and might resolve problems that would have caused high mental workload. The strong associations with interruptions from colleagues provide preliminary support for this idea.

The overall volume of workflow interruptions showed best prediction in workload, supporting the notion that multilayered disruptions to clinical work are likely to have an effect significantly more pronounced than the effect of individual distracting events [20].

### Limitations of the study

Several limitations of our study deserve consideration. The first and main limitation was the lack of check for the content of the interruptive events. Although we assessed the source of the interruption we cannot infer about the events’ potential for complexity and demands in clinical decision making. In our study, only observable, pre-defined workflow disruptions were recorded. But, interruptions may be valuable for fast-responding clinical care and different interruptions may have different subjective effects and clinical implications [25, 34, 35]. Further studies should take a closer look at the content of interruptions. Secondly, doctors’ individual perception of interruptive events may differ substantially according to severity, temporal duration, or nature of the event [2, 36]. Pediatricians’ working memory capacity could also be relevant, such that physicians with greater memory capacity may be less susceptible to interruptions [24, 37]. Thirdly, the study’s prospective design is a feasible way to address prospective associations of interruptions and workload outcomes, but fails to establish causality. To infer causality controlled intervention studies in applied or simulation settings are needed [14]. Fourth, results are based on a convenience sample of a children hospital in Germany. Although we checked for comparability, we cannot exclude the possibility that patient census, clinical demands, and associated workload are different in non-academic hospital settings. All observations covered pediatricians’ ward work and we acknowledge that the level of interruptions may be different within other clinical areas, e.g., emergency care or intensive care units [30, 33].

### Implications of the study

The study results suggest various clinical and research implications. Clinically, addressing and reducing unnecessary workflow interruptions is a feasible way to improve pediatricians’ work life [20, 38]. Specifically, interventions to limit irrelevant interruptions may reduce pediatricians’ frustration and strain. Our findings also suggest that intervention approaches for improving the communication among the unit staff would be advantageous. These improvements could be accomplished through enhancement of inter-professional collaboration, better organization of various tasks, deliberate design of joint activities and more efficient information transfer [33, 39, 40]. We suggest team-based interventions to identify and reduce unnecessary interruptions in order to limit overall ‘interruptibility’ in pediatricians work [41]. Interventions should aim to design ‘interruption resilient work’ for hospital pediatricians. The inherent challenge will be to maintain inevitable, necessary interruptions that support clinical workflow, collaboration, and contribute to safety; as well as to smoothen workflow through reducing unnecessary, ineffective interruptions that have detrimental consequences [25]. Potential beneficial effects for less interruptive workflow and feasible workload should be evaluated [30, 40, 41].

Potential research implications include the differential impact of diverse interruption sources to different workload outcomes. Future studies should focus on potential costs and detrimental effects on pediatricians’ efforts of regularly working in highly interruptive, stress-inducing environments, i.e., stress, fatigue, or failures at work. More elaborate research designs that investigate the impact of interruptions on stress alongside individual doctors’ compensatory behaviors could stimulate interventions in work design and training approaches in pediatric care. Aside, a further possibility could be to identify and train effective behavioral strategies to cope successfully with interruptions at the clinical workplace. Our study captured potential cognitive effects of frequent workflow disruptions and therefore provides ground to create an effective cognitive environment for healthcare workers [24].

## Conclusions

Our study found a detrimental association of workflow interruptions on pediatrician reported frustration and stress. Concurrently, observed interruptions were negatively associated with pediatricians’ mental demands. Our findings support previous research addressing potential negative effects of workflow interruption on clinicians’ stress, the efficiency of clinical work, as well as patient safety [14, 25]. In line with system approaches in patient safety we suggest that pediatricians’ work environment needs to be designed in terms of socio-technical systems that balance human-human and human-technology interactions, i.e., aligning human-oriented re-design-efforts with needs for effective and safe functioning of healthcare delivery [25]. It calls for well-designed intervention strategies that reduce ineffective and redundant interruptions by maintaining necessary, inevitable disruptions that contribute to patient safety and clinical collaboration [14, 25, 26].

## References

[1] Laxmisan A, Hakimzada F, Sayan OR, Green RA, Zhang J, Patel VL (2007). The multitasking clinician: decision-making and cognitive demand during and after team handoffs in emergency care. Int J Med Inform.

[2] Sevdalis N, Forrest D, Undre S, Darzi A, Vincent C (2008). Annoyances, disruptions, and interruptions in surgery: the Disruptions in Surgery Index (DiSI). World J Surg.

[3] Westbrook JI, Ampt A, Kearney L, Rob MI (2008). All in a day’s work: an observational study to quantify how and with whom doctors on hospital wards spend their time. Med J Aust.

[4] Chisholm CD, Dornfeld AM, Nelson DR, Cordell WH (2001). Work interrupted: a comparison of workplace interruptions in emergency departments and primary care offices. Ann Emerg Med.

[5] Antoniadis S, Passauer-Baierl S, Baschnegger H, Weigl M (2014). Identification and interference of intraoperative distractions and interruptions in operating rooms. J Surg Res.

[6] Walter SR, Li L, Dunsmuir WT, Westbrook JI (2014). Managing competing demands through task-switching and multitasking: a multi-setting observational study of 200 clinicians over 1000 hours. BMJ Qual Saf.

[7] Weigl M, Müller A, Sevdalis N, Angerer P (2013). Relationships of multitasking, physicians’ strain, and performance: an observational study in ward physicians. J Patient Saf.

[8] O’Shea E (1999). Factors contributing to medication errors: a literature review. J Clin Nurs.

[9] Westbrook JI, Coiera E, Dunsmuir WT, Brown BM, Kelk N, Paoloni R, Tran C (2010). The impact of interruptions on clinical task completion. Qual Saf Health Care.

[10] Westbrook JI, Woods A, Rob MI, Dunsmuir WT, Day RO (2010). Association of interruptions with an increased risk and severity of medication administration errors. Arch Intern Med.

[11] Wiegmann DA, ElBardissi AW, Dearani JA, Daly RC, Sundt TMI (2007). Disruptions in surgical flow and their relationship to surgical errors: an exploratory investigation. Surgery.

[12] Hakimzada AF, Green RA, Sayan OR, Zhang J, Patel VL (2008). The nature and occurrence of registration errors in the emergency department. Int J Med Inform.

[13] Jett QR, George JM (2003). Work interrupted: a closer look at the role of interruptions in organizational life. Acad Manage Rev.

[14] Coiera E (2012). The science of interruption. BMJ Qual Saf.

[15] Hart SG, Staveland LE, Hancock PA, Meshkati N (1988). Development of NASA-TLX (Task Load Index): Results of Empirical and Theoretical Research. Human Mental Workload.

[16] Bertram DA, Opila DA, Brown JL, Gallagher SJ, Schifeling RW, Snow IS, Hershey CO (1992). Measuring physician mental workload: reliability and validity assessment of a brief instrument. Med Care.

[17] Byrne AJ, Oliver M, Bodger O, Barnett WA, Williams D, Jones H, Murphy A (2010). Novel method of measuring the mental workload of anaesthetists during clinical practice. Brit J Anaesth.

[18] Holden RJ, Scanlon MC, Patel NR, Kaushal R, Escoto KH, Brown RL, Alper SJ, Arnold JM, Shalaby TM, Murkowski K, Karsh BT (2011). A human factors framework and study of the effect of nursing workload on patient safety and employee quality of working life. BMJ Qual Saf.

[19] Weigl M, Müller A, Vincent C, Angerer P, Sevdalis N (2012). The association of workflow interruptions and hospital doctors’ workload: a prospective observational study. BMJ Qual Saf.

[20] Sevdalis N, Sonal A, Undre S, Vincent CA, Flin R, Mitchell L (2009). Distractions and Interruptions in the Operating Room. Safer Surgery: Distractions and Interruptions in the Operating Room.

[21] Tucker AL, Spear SJ (2006). Operational failures and interruptions in hospital nursing. Health Serv Res.

[22] Montgomery VL (2007). Effect of fatigue, workload, and environment on patient safety in the pediatric intensive care unit. Pediatr Crit Care Med.

[23] Gabow PA, Karkhanis A, Knight A, Dixon P, Eisert S, Albert RK (2006). Observations of residents’ work activities for 24 consecutive hours: implications for workflow redesign. Acad Med.

[24] Grundgeiger T, Sanderson P (2009). Interruptions in healthcare: theoretical views. Int J Med Inform.

[25] Rivera-Rodriguez AJ, Karsh BT (2010). Interruptions and distractions in healthcare: review and reappraisal. Qual Saf Health Care.

[26] Li SY, Magrabi F, Coiera E (2012). A systematic review of the psychological literature on interruption and its patient safety implications. J Am Med Inform Assoc.

[27] Hart SG (2006). Nasa-task load index (Nasa-TLX); 20 years later. Proc Hum Factors Ergon Soc Annu Meet.

[28] Weigl M, Hoffmann F, Muller A, Barth N, Angerer P (2014). Hospital paediatricians’ workflow interruptions, performance, and care quality: a unit-based controlled intervention. Eur J Pediatr.

[29] Weigl M, Müller A, Zupanc A, Glaser J, Angerer P (2011). Hospital doctors’ workflow interruptions and activities: an observation study. BMJ Qual Saf.

[30] France DJ, Levin S, Hemphill R, Chen K, Rickard D, Makowski R, Jones I, Aronsky D (2005). Emergency physicians’ behaviors and workload in the presence of an electronic whiteboard. Int J Med Inform.

[31] Greiner BA, Ragland DR, Krause N, Syme SL, Fisher JM (1997). Objective measurement of occupational stress factors-an example with San Francisco urban transit operators. J Occup Health Psychol.

[32] Levin S, France DJ, Hemphill R, Jones I, Chen KY, Rickard D, Makowski R, Aronsky D (2006). Tracking workload in the emergency department. Hum Factors.

[33] Mache S, Vitzthum K, Kusma B, Nienhaus A, Klapp BF, Groneberg DA (2010). Pediatricians’ working conditions in German hospitals: a real-time task analysis. Eur J Pediatr.

[34] Brixey JJ, Robinson DJ, Turley JP, Zhang J (2010). The roles of MDs and RNs as initiators and recipients of interruptions in workflow. Int J Med Inform.

[35] Kreckler S, Catchpole K, Bottomley M, Handa A, McCulloch P (2008). Interruptions during drug rounds: an observational study. Br J Nurs.

[36] Healey AN, Sevdalis N, Vincent CA (2006). Measuring intra-operative interference from distraction and interruption observed in the operating theatre. Ergonomics.

[37] Trafton JG, Monk CA (2007). Task interruptions. Rev Hum Factors Ergon.

[38] Zijlstra FRH, Roe RA, Leonora AB, Krediet I (1999). Temporal factors in mental work: effects of interrupted activities. J Occup Organ Psychol.

[39] Patterson ES, Roth EM, Woods DD, Chow R, Gomes JO (2004). Handoff strategies in settings with high consequences for failure: lessons for health care operations. Int J Qual Health Care.

[40] Catchpole K, Ley E, Wiegmann D, Blaha J, Shouhed D, Gangi A, Blocker R, Karl R, Karl C, Taggart B, Starnes B, Gewertz B (2014). A human factors subsystems approach to trauma care. JAMA Surg.

[41] Weigl M, Hornung S, Glaser J, Angerer P (2012). Reduction of hospital physicians’ workflow interruptions: a controlled unit-based intervention study. J Healthc Eng.

[CR42] The pre-publication history for this paper can be accessed here:http://www.biomedcentral.com/1472-6963/14/433/prepub

